# Pre-Surgical Factors Influencing Post-Surgical Outcomes in Orthognathic Surgery Patients: A Longitudinal Study

**DOI:** 10.3390/jcm13154445

**Published:** 2024-07-29

**Authors:** Gonzalo Navarro-Fernández, Javier Bravo-Aparicio, Jose Luis Del Castillo, Hector Beltran-Alacreu, Alfonso Gil-Martínez

**Affiliations:** 1 Escuela Internacional de Doctorado, Department of Physical Therapy, Occupational Therapy, Rehabilitation and Physical Medicine, Universidad Rey Juan Carlos, 28922 Alcorcón, Spain; gnavfe@gmail.com; 2 CranioSpain Research Group, Centro Superior de Estudios Universitarios La Salle, Universidad Autónoma de Madrid, 28043 Madrid, Spain; alfonso.gil@lasallecampus.es; 3 Toledo Physiotherapy Research Group (GIFTO), Faculty of Physical Therapy and Nursing, Universidad de Castilla-La Mancha, 45071 Toledo, Spain; javier.bravo@uclm.com; 4 Instituto de Investigación Sanitaria de Castilla-La Mancha (IDISCAM), 45071 Castilla-La Mancha, Spain; 5 Department of Oral and Maxillofacial Surgery, University Hospital La Paz, 28046 Madrid, Spain; jldelcastillo@gmail.com; 6 Departamento de Fisioterapia, Centro Superior de Estudios Universitarios La Salle, Universidad Autónoma de Madrid, 28043 Madrid, Spain; 7 Unit of Physiotherapy, Hospital La Paz-Carlos III, Institute for Health Research IdiPAZ, 28046 Madrid, Spain

**Keywords:** orthognathic surgery, pre-surgical factors, post-surgical evolution, range of motion, anxiety, kinesiophobia

## Abstract

**Background/Objectives**: This study aims to assess the relationship between physical and psychosocial pre-surgical factors and post-surgical evolution in patients undergoing orthognathic surgery. **Methods**: A cohort study with 3 months of follow-up after maxillofacial surgery was conducted. Participants were recruited from the Maxillofacial Surgery Unit of Hospital Universitario La Paz in Madrid, Spain. Primary variables included the range of motion of mouth opening, protrusion tongue force, anxiety, depression and kinesiophobia. Assessments were realised on-site or via video call. Statistical analysis was conducted using mixed-effects models. **Results**: The initial recruitment yielded 22 patients, with 19 ultimately eligible for analysis. The study found significant impacts of pre-surgical factors on post-surgical evolution. Both ranges of motion and anxiety showed influences from baseline measures, with the range of motion affected by a pre-surgical range of motion (estimate: 3.89) and positive expectations (estimate: 4.83). Anxiety was influenced by both pre-surgical (estimate: 0.48) and baseline anxiety levels (estimate: 0.64). Kinesiophobia demonstrated a trend toward significance, with baseline levels affecting post-surgical evolution (estimate: 0.77). **Conclusions**: Our results highlight the relationship between pre-surgical factors and post-surgical outcomes in orthognathic surgery patients. Pre-surgical range of motion and positive expectations were found to influence post-surgical range of motion, while pre-surgical anxiety levels impacted post-surgical anxiety evolution. Pre-surgical kinesiophobia also showed potential as a post-surgical kinesiophobia predictor, but further investigation is needed to confirm this relationship.

## 1. Introduction

Orthognathic surgery is recommended for the correction of moderate or severe dentofacial deformities and occlusion problems, with the objective of achieving balance and facial proportion in all planes of the patient’s face, as well as correcting functionality [1]. According to a study published in 2014, 52% of orthognathic procedures are conducted due to functional impairments (i.e., occlusion problems or sleep apnoea), while 27% of the cases are related to aesthetic reasons (i.e., facial asymmetry) [2].

Something that is intrinsically related to any surgical procedure is the presence of adverse effects and its management. Even though the most severe adverse effects are infrequent after an orthognathic surgery [3], there are some complications that should be considered because of their influence on the patient’s recovery. For example, neurological sequelae due to cranial nerve injuries (usually trigeminal nerve or facial nerve) can produce sensory disorders in more than 15% of patients over 30 years of age who undergo bilateral sagittal mandibular osteotomy [4]. In another similar study, the authors observed that more than 60% of patients undergoing orthognathic surgery have paraesthesia or dysesthesia that lasts up to 6 months after the surgery [5], and these symptoms can condition orofacial function [6]. An additional complication that warrants consideration is post-surgical pain. A study conducted by Agbaje et al. in 2018 investigated pain complaints associated with this type of surgery. Their findings revealed that nearly 18% of patients continued to experience pain one year post-surgery [7].

Furthermore, the psychosocial aspect of patients undergoing orthognathic surgery is a significant factor. It has been noted that these patients exhibit higher levels of anxiety compared to the general population [8]. Some studies have thus concluded that it would be beneficial to evaluate the mental health status of patients prior to orthognathic surgery [9]. Also, in relation to psychosocial factors and surgical procedures, a meta-analysis has been published that affirms that patients’ expectations of the results significantly impact their post-surgical quality of life. The authors found this to be consistent across various types of surgery [10]. For instance, it was observed that patients who underwent a rhinoplasty with pre-existing nasal asymmetry were often less satisfied with the surgical result, likely due to unrealistic expectations [11].

While there are studies that focus on conservative treatments to reduce post-surgical complications in orthognathic and oral surgeries [12,13,14], to the authors’ knowledge, no studies have addressed these issues at the pre-surgery stage. When examining other surgery procedures, several meta-analytic studies recommend prehabilitation programs to improve the post-surgical outcomes and patient quality of life, as seen in major abdominal surgery [15] and orthopaedic surgery [16]. However, no comparable studies exist for orthognathic surgery. In fact, we lack certainty about which pre-surgical factors are related to post-surgical prognosis in orthognathic surgery. Even though there are some modern imaging techniques that are being used for surgery planning, their relationship with surgical outcomes and prognosis is not clear yet [17]. Thus, it is necessary to start addressing the issue of pre-surgical factors related to surgery prognosis to identify potential therapeutic targets to develop prehabilitation programs in orthognathic surgery.

Consequently, the objective of the present study is to evaluate the relationship between physical and psychosocial pre-surgical factors and the post-surgical evolution of patients undergoing orthognathic surgery.

## 2. Materials and Methods

### 2.1. Study Design

This research was a prospective cohort study with a follow-up period of 3 months post-maxillofacial surgery. The procedures used in this research project were conducted under the guidelines of the Helsinki Declaration. All study participants were required to give their written informed consent prior to the start of the research, which was previously approved by the Ethics Committee from the Hospital Universitario La Paz (project code: PI-4943). The study was conducted following the STROBE statement for observational studies [18].

### 2.2. Setting and Participants

The sample was drawn from the Maxillofacial Surgery Unit of the Hospital Universitario La Paz in Madrid, Spain. Enrollment began in February 2022 and finished in February 2024. Patients recruited in this study were men and women aged between 18 and 65 years who were scheduled for maxillofacial surgery. The authors considered that patients under 18 or above 65 years old could have surgical outcomes that could be less representative of the general population, so they were excluded. Patients with systemic illnesses such as cancer or multiple sclerosis were also excluded from the study, as the symptoms of other important pathologies could influence the evolution of maxillofacial surgery. This exclusion criterion was implemented to ensure a more homogeneous sample and to focus on the effects of the surgery itself rather than potential complications from other health conditions. Patients were measured 6 times, twice before surgery and 4 times after surgery in a 3-month follow-up.

### 2.3. Outcomes

The primary outcomes assessed were the range of motion (ROM) of mouth opening, tongue force, anxiety, depression and kinesiophobia.

Range of Motion of mouth opening. The ROM was assessed with the Therabite scale, a disposable cardboard tool designed to measure the mandibular range of motion in millimetres and validated for self-measurement processes [19].

Tongue force. The protrusion tongue force was assessed with a new, less-invasive device composed of an FSR sensor, an Arduino microprocessor and software for the measurements. This tool has been demonstrated to be a valid and reliable tool for tongue force measurements [20,21]. Considering the surgical scenario of the patients, this tool was selected because it was less invasive than other options.

Depression and Anxiety. Both anxiety and depression were assessed with the Hospital Anxiety and Depression Scale (HADS). This scale is composed of 14 items (7 for anxiety and 7 for depression); each of them is valued with 4 possible answers ranging from 0 to 3 points. The final score varies from 0 to 21 for both anxiety and depression. This scale has been translated into Spanish, showing good psychometric properties (Cronbach’s alpha = 0.86) [22].

Kinesiophobia. The fear of movement or kinesiophobia was assessed with the Tampa Scale of Kinesiophobia (TSK-11), composed of 11 Likert-like items punctuated from 1 to 4. The scores range between 11 and 44, with higher scores related to higher levels of kinesiophobia. This scale is widely used and has also been validated in Spanish with an acceptable internal consistency (Cronbach’s alpha = 0.79) [23].

### 2.4. Secondary Outcomes

In addition to these primary variables, other variables were assessed. The pain intensity was measured with a numeric scale, which has been accepted as a good option for pain measurements [24]. The expectations of the patients with surgery and recovery were measured with an adapted version of the Stanford Expectations of Treatment Scale (SETS). This scale is composed of 6 items and assesses positive expectations (3 items) and negative expectations (3 items). It has been validated in English [25] but not in Spanish, so the authors created an ad hoc version for this study. The quality of life was measured with the Orthognathic Quality of Life Questionnaire (OQLQ), which has 22 items divided into 4 domains (facial aesthetics, oral function, asymmetry worry and social aspects). This tool has been validated and adapted into Spanish [26]. Finally, other variables related to the surgery were recorded as time on a waiting list, surgery time or hospitalisation days.

Regarding other variables, for mixed-effects model analysis, both the age and body mass index (BMI) were considered as covariates as both could condition the result of the analysis.

### 2.5. Biases

As explained before, Therabite is a tool validated for the self-measurement of ROM. To avoid biases during the measurements and to ensure that the self-measurements performed at home were reliable, the researchers trained and supervised the patients during the first measurements. These training measurements were not recorded. Once the researchers considered that the patients were able to correctly measure their mandibular ROM and that the real measurements were started, in order to avoid biases, the researchers did not see the score directly on the scale but recorded the score the patients referred.

### 2.6. Procedure

Patients were recruited from the waiting list of the hospital’s Maxillofacial Surgery Unit. There were 6 observations per patient. For the first one, the researchers contacted each subject, and if they agreed to participate in the study, they were scheduled for an on-site evaluation. In this evaluation, the researchers informed the patients about the whole process of the study, and informed consent was obtained. Afterward, the researchers gathered some information such as evaluation date; age; gender; height; weight; and surgery-related information such as medical diagnosis, time since diagnosis or waiting list time. After this interview, the patients were trained to perform a self-evaluation of ROM using the Therabite, and the researchers gave them an informative sheet about the ROM measurement process and some Therabite scales so that they could measure ROM at home (Appendix A). This variable was recorded 3 times per observation, and the average was computed later. The researchers also evaluated the protrusion tongue force of the patient. In this case, as the measure was for maximum protrusion tongue force, in order to avoid fatigue by repeating the assessment, it was performed only once per observation, and the fatigue was recorded after it using a numerical scale from 0 to 10. Finally, the patient fulfilled the required questionnaires. If this first evaluation was not performed between 1 and 7 days before the surgery, it was considered a baseline evaluation, and the patient was scheduled for a second evaluation in the 1 to 7 days before the surgery. This second evaluation was considered pre-surgical evaluation, and the procedure was the same, except for the informed consent, which was only signed once.

After the surgery, the patients were followed up 4 times: 2 weeks after the surgery, 4 weeks after the surgery, 8 weeks after the surgery and 12 weeks after the surgery. All these evaluations were performed remotely using a video call platform (Microsoft Teams), except for the last one, which was on-site again. During the video call interviews, the information was gathered by the researchers to ensure its reliability. This information was related to the surgery evolution, such as pain intensity, presence of adverse effects or received treatments, and the ROM, which was self-measured again following the researchers’ instructions and the training performed in the first session. Then, the patients fulfilled the questionnaires. During the last on-site evaluation, the procedure was the same, but the tongue force was measured again.

There was no modification of any treatment any patient was receiving before or after the surgery.

### 2.7. Sample Size Calculation

In relation to the primary outcomes, the sample size was initially estimated for a total of five possible predictors. According to Cohen’s guidelines [27], it was assumed that a large effect size (ƒ^2^ = 0.35) for the multiple regression model. Thus, considering an alpha error of 0.05 and a power of 0.80, a total sample size of 20 subjects was obtained. Considering the follow-up period and the dropout risk, the sample size was increased by 10%, resulting in 22 subjects. The software used for the sample size calculation was G*Power Program 3.1.9.2 from the University of Düsseldorf [28].

### 2.8. Statistical Analysis

The statistical analysis was performed using RStudio (2023.03-0+386 version, Posit Software, PBC) statistical software.

1. Demographic data

Demographic data were presented as median and inter-quartile range and histograms. The Shapiro–Wilk test for normal distribution was performed in demographic data to assess for normality.

2. General evolution analysis.

For each of the primary variables, a mixed-effects analysis was performed, considering time as a fixed effect and intra-subject variability as a random effect. Age and BMI were used as covariables.

The general formula of mixed-effects models is

$$y_{ij}=\beta_{0}+\beta_{1}x_{1ij}+\beta_{2}x_{2ij}+\ldots +\beta_{n}x_{nij}+b_{i0}+b_{i1}z_{1ij}+\ldots +b_{in}z_{nij}+\varepsilon_{ij}$$

where $y_{ij}$ is the dependent variable, $\beta_{n}$ are the coefficients of fixed effects used as predictors ($x_{nij}$), $b_{in}$ are the coefficients of the random effects used as predictors ($z_{nij}$), and $\varepsilon_{ij}$ is the random error.

The goal of this analysis is to observe if the time or the intercept ($\beta_{0}$, equivalent to the baseline, when there are no fixed effects) have a significant impact on the dependent variable evolution, considering all observations.

3. Pre-surgical variables impact on post-surgical evolution analysis

From previous step models, for those where a significant result was obtained, a secondary mixed-effects analysis was carried out, adding baseline and pre-surgical observations as predictors.

To avoid overfitting in the models, we applied the rule of 10 observations of the dependent variable per predictor in the model [29]. For those models where we had more predictors than allowed, we followed an iterative selection process, adding and removing predictors to the model. The resulting models were compared using the Bayesian Information Criteria (BIC)

$$BIC=-2\log\left(L\right)+klog(n)$$

where *L* is the model’s likelihood, *k* is the number of predictors introduced in the model, and *n* is the sample size. The BIC is considered one of the best indicators for comparing the fitting of different linear models with multiple variables, following the criteria that the lower the BIC, the better the fit of the model [30].

## 3. Results

**Figure 1:**
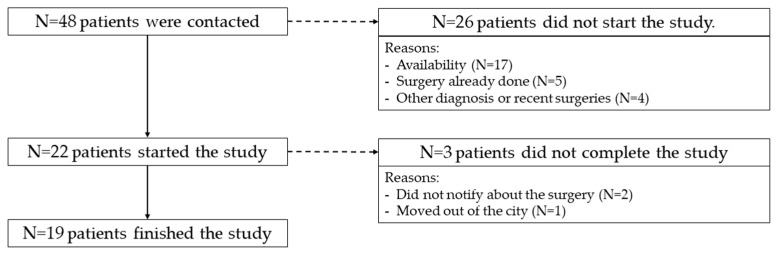
Flow diagram.

**Figure 2:**
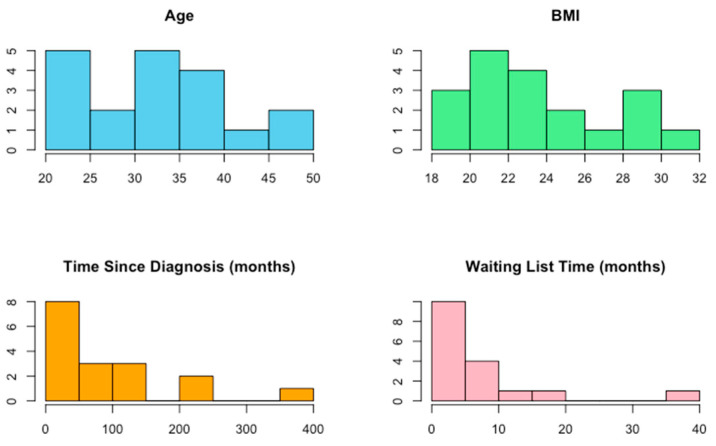
Histograms.

Initially, 22 patients were recruited for the study. However, 1 patient moved to another city, and 2 patients did not advise of the surgery date, so only 19 were finally eligible for the analysis (16 women, 3 men). Figure 1 represents the flow diagram. The demographics and Shapiro test analysis are represented in Table 1 and Figure 2.

### 3.1. General Evolution

**Figure 3:**
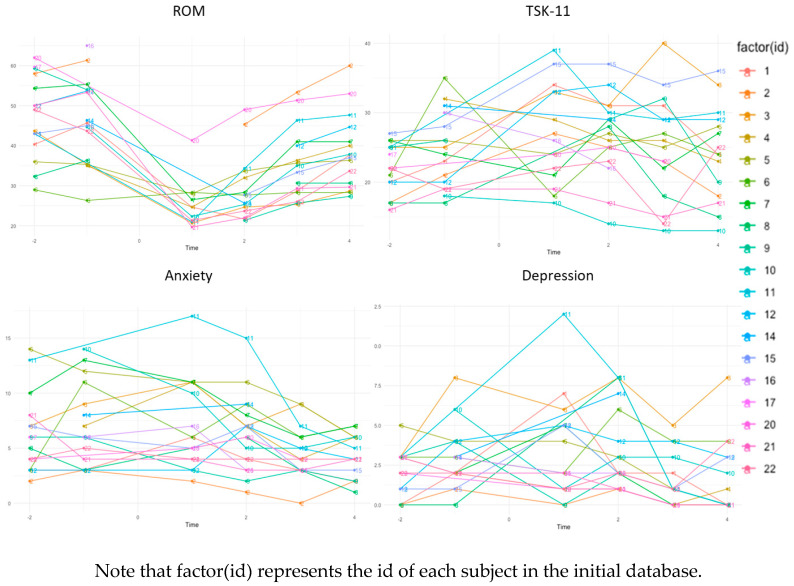
Evolution charts.

Evolution charts for ROM, kinesiophobia, anxiety and depression are shown in Figure 3. For ROM as dependent variable, both the intercept (estimate: 31.88; SE: 14.67; 95% CI: from 4.00 to 59.51) and time (estimate: −1.97; SE: 0.43; 95% CI: from −2.83 to −1.14) were statistically significant. For the rest of the primary outcomes, the results were statistically significant only in anxiety and kinesiophobia. For anxiety, both the intercept (estimate: 9.03; SE: 4.74; 95% CI: from 0.04 to 18.03) and the time (estimate: −0.45; SE: 0.11; 95% CI: from −0.66 to −0.24) were significant, while for kinesiophobia, it was significant only the intercept (estimate: 29.29; SE: 8.07; 95% CI: from 13.97 to 44.58). All the results of this general evolution analysis are summarised in Table 2.

### 3.2. Baseline and Pre-Surgical Variables Impact on Post-Surgical Evolution

For ROM evolution, the changeover post-surgical evaluations were statistically significative (estimate: 3.89; SE: 0.40; 95% CI: from 3.12 to 4.70) and a statistically significant effect was observed for pre-surgical ROM evaluation (estimate: 0.35; SE: 0.18; 95% CI: from 0.04 to 0.65) and pre-surgical positive expectation (estimate: 4.83; SE: 2.23; 95% CI: from 1.07 to 8.60). For kinesiophobia evolution, the changeover post-surgical evaluations were not statistically significant, but a statistically significant effect was observed for baseline kinesiophobia evaluation (estimate: 0.77; SE: 0.35; 95% CI: from 0.15 to 1.39). Both ROM and kinesiophobia results are summarised in Table 3.

For anxiety evolution, two models were created: one considering pre-surgical evaluation and another considering baseline evaluation. Both are summarised in Table 3. In both cases, the changeover post-surgical evaluations were statistically significant. For the pre-surgical model, it was observed a statistically significant effect of pre-surgical anxiety (estimate: 0.48; SE: 0.11; 95% CI: from 0.28 to 0.68), while for the baseline model, statistically significant effects were found for baseline anxiety (estimate: 0.64; SE: 0.13; 95% CI: from 0.41 to 0.87) and age (estimate: 0.13; SE: 0.06; 95% CI: from 0.02 to 0.24).

## 4. Discussion

This study shows that for both ROM and anxiety, the general evolution is not only influenced by the time factor: the individual baseline situation of the subjects also had a statistically significant impact on the dependent variable evolution. This means that the time itself is not enough to explain how the ROM and anxiety evolve in each of the observations, and the starting point of each patient has a significant impact on the model. By isolating the baseline and pre-surgical measurements, we can take a closer look at this relationship.

The first thing that we can see with the models described in Table 3 is that the intercept is no longer significant, meaning that the starting point after the surgery is mostly the same for all subjects. For the ROM model, we found that there were two different factors that could act as predictors of post-surgical evolution: pre-surgical ROM and pre-surgical positive expectations. These results can be interpreted as follows: for each unit in pre-surgical ROM, an increase of 0.35 units in post-surgical ROM is expected, and for each unit in pre-surgical positive expectations, an increase of 4.83 units in post-surgical ROM is expected. The 0.35 change in ROM could be interpreted as a less relevant result considering previous studies’ results. It has been shown that the standard error of measurement (SEM) of maximum mouth opening using the Therabite tool was between 0.68 and 0.92 [31]. However, in the cited article, the measurement was performed by the researchers instead of by the patients themselves, and the patients had a diagnosis of temporomandibular disorders, meaning that the results could not be comparable with the results obtained in the current study. Additionally, Beltrán-Alacreu et al. performed another study in 2014 with asymptomatic subjects obtaining similar values for SEM (from 0.74 to 0.82). However, in this study, the tool used to measure mandibular ROM was not Therabite, so the results are also not comparable with those obtained in this article [32]. So, even though the 0.35 estimate obtained seems to be less relevant considering the SEM obtained previously, we cannot be completely sure that these results are comparable with ours.

The other relevant factor related to post-surgical ROM evolution is pre-surgical positive expectations measured with the SETS scale. In this case, the estimate is 4.83, meaning a greater impact on ROM evolution. The influence of patients’ expectations on post-surgical evolution has been investigated previously. In the study of Al-Hadi et al., published in 2019, even though the authors used a different way to assess the patients’ expectations, they found interesting results. They considered that the surgery satisfaction was related to meeting expectations and concluded that one area of improvement was information delivery before the surgery [33]. They found that around 17% of the patients did not receive information about important aspects of the surgical procedure, such as the duration, and around 8% did not feel informed about the complications of the procedure. This can influence how the patients perceive the surgery outcomes, so it is important to provide as much information as possible before the surgery with the objective of helping patients set realistic expectations. These findings are also present in other surgery types. In a meta-analysis of 46 studies about total hip and knee arthroplasty, a robust small association between pre-operative positive expectations and post-operative outcomes was found [34]. In our results, the relation between expectations and post-surgery evolution is also observed, and we now have a numeric estimation of how big this relation could be. Improving one point in the SETS punctuation means potentially increasing more than four units of ROM measured with the Therabite.

For the anxiety model, we also obtained statistically significant results for different factors. As anxiety had multiple possible predictors, we needed to create two different models: one focused on baseline observation variables and another focused on pre-surgical observation variables. Considering baseline observation variables, we found two possible predictors for the post-surgery anxiety evolution: baseline anxiety and age. On the other side, considering pre-surgery observation variables, only pre-surgical anxiety was found to be a statistically significant predictor of post-surgery anxiety evolution. The fact that the age was significant only for baseline observations could be explained as, at baseline, greater age is related to post-surgical anxiety, but the closer the surgery date, the lower the difference by age. In any case, both the baseline and pre-surgical anxiety levels are related to anxiety post-surgery evolution. It is important to consider pre-surgical anxiety level as it is associated with quality of life [35] and with other post-operative symptoms [36]. There are articles that have studied the presence of anxiety in orthognathic surgery patients and its causes. In a recent study conducted by Kok et al. in 2023, they found that one of the factors related to reduced anxiety levels was the satisfaction of the patients with the information provided by the clinical team [37], and this relation between anxiety and knowledge has been found in previous articles [38].

Finally, the kinesiophobia model showed a tendency to have a significant effect on time (the upper limit of the confidence interval was slightly superior to 0). However, the statistically significant impact of baseline kinesiophobia on post-surgical kinesiophobia evolution might make this variable a possible pre-surgical treatment target, but further research is needed.

The results obtained in the present study show that the pre-surgical status of patients who undergo maxillofacial surgery plays a significant role in post-surgical outcomes. Specifically, pre-surgical ROM and expectations influence the post-surgical evolution of ROM, while pre-surgical anxiety influences post-surgical anxiety evolution. Their findings highlight the importance of a pre-surgical multidisciplinary assessment in order to improve the prognosis of the patients. Based on our results, factors such as ROM or anxiety could be used as therapeutic targets for pre-surgical treatments that might reduce post-surgical complications and improve patients’ recovery, as these results suggest that the better these pre-surgical factors, the better the post-surgical evolution. However, we recommend taking these results with caution, considering that the present study has some limitations. First, the sample size can be considered reduced, and the lack of a control group could make the results of the present study less comprehensive. Second, the SETS questionnaire has not been validated in Spanish, so our results in this variable could be influenced by this condition. Third, as explained before, the results obtained for pre-surgery ROM impact on post-surgical ROM should be taken with caution as the amount could be under the SEM limit found in other studies. Fourth, we have not considered the use of diagnostic images as pre-surgical variables due to limited resources. Finally, even though the researchers did not register any problem and actions were performed to mitigate any negative impact, using video call platforms can introduce unexpected variability in data that should be considered a potential limitation.

## 5. Conclusions

Our results highlight the relationship between pre-surgical factors and predicting post-surgical outcomes in orthognathic surgery patients. Pre-surgical range of motion and positive expectations were found to influence post-surgical range of motion, while pre-surgical anxiety levels impacted post-surgical anxiety evolution. Even though the effect of a pre-surgical range of motion effect is reduced, its relationship with a post-surgical range of motion evolution was found statistically significant, and it should be taken into consideration. Another important factor to consider in the pre-surgical scenario is the assessment of the anxiety levels of the patients and their expectations with the surgery, as both can turn into worse post-surgical evolution. Pre-surgical kinesiophobia also showed potential as a post-surgical kinesiophobia predictor, but further investigation is needed to confirm the relationship. The results and limitations of this study suggest that a multidisciplinary approach is needed in the next investigations, considering clinical, imaging and patient-specific factors.

## Figures and Tables

**Table 1:** Demographics and normality test.

Variable	Median	IQ Range (Q3-Q1)	*p*-Value Shapiro–Wilk
Age	32	13 (39–26)	*p* = 0.38
BMI	22.86	5.32 (25.68–20.36)	*p* = 0.05
Evolution time in months (since diagnosis)	60	88 (112–24)	*p* < 0.01
Waiting list time in months	5	6 (9–3)	*p* < 0.01

IQ: Interquartile range; BMI: body mass index.

**Table 2:** Main outcomes general evolution analysis.

	*ROM*			*TF Max*			*TF Avg*		
***Fixed Effects***	***Est.***	***SE***	***95% CI***	***Est.***	***SE***	***95% CI***	***Est.***	***SE***	***95% CI***
*Intercepct*	31.88	14.67	4.00 to 59.51 *	20.01	17.34	−12.75 to 52.72	15.07	12.77	−9.08 to 39.14
*Time*	−1.97	0.43	−2.83 to −1.14 *	0.66	0.36	−0.08 to 1.39	0.43	0.25	−0.08 to 0.93
*Age*	−0.09	0.27	−0.60 to 0.43	−0.19	0.32	−0.79 to 0.41	−0.09	0.23	−0.53 to 0.35
*BMI*	0.51	0.60	−0.61 to 1.65	0.11	0.68	−1.17 to 1.39	−0.07	0.50	−1.01 to 0.87
***Random Effects***	**Std Dev**			**Std Dev**			**Std Dev**		
*Subjects (intercept)*	7.73			9.53			7.11		
	**TSK11**			**HADS Anxiety**			**HADS Depression**		
***Fixed Effects***	***Est.***	***SE***	***95% CI***	***Est.***	***SE***	***95% CI***	***Est.***	***SE***	***95% CI***
*Intercept*	29.29	8.07	13.97 to 44.58 *	9.03	4.74	0.04 to 18.03 *	1.70	2.49	−3.00 to 6.43
*Time*	0.36	0.21	−0.07 to 0.77	−0.45	0.11	−0.66 to −0.24 *	−0.13	0.10	−0.33 to 0.07
*Age*	0.15	0.15	−0.13 to 0.44	0.03	0.09	−0.14 to 0.19	0.08	0.05	−0.01 to 0.16
*BMI*	−0.41	0.33	−1.03 to 0.21	−0.14	0.19	−0.50 to 0.22	−0.06	0.10	−0.26 to 0.13
***Random Effects***	**Std Dev**			**Std Dev**			**Std Dev**		
*Subjects (intercept)*	4.42			2.67			1.15		

Est.: estimate; SE: standard error; 95% CI: 95% confidence interval; Std Dev.: standard deviation; BMI: body mass index; ROM: range of movement; TF Max: Maximum Tongue Force; TF Avg: Tonge Force Average; TSK11: Tampa Scale of Kinesiophobia; HADS: Hospital Anxiety and Depression Scale. (*) indicates *p*-values lower than 0.05.

**Table 3:** 1. Pre–post models for ROM and TSK. 2. Pre–post models for anxiety: one with pre-surgical variables and one with baseline variables.

**1**							
	ROM (1)				TSK 11 (2)		
**Fixed Effects**	*Est.*	*SE*	*95% CI*	**Fixed Effects**	*Est.*	*SE*	*95% CI*
Intercept	−28.98	20.74	−64.07 to 6.03	Intercept	22.00	13.73	−2.39 to 46.42
Time	3.89	0.40	3.12 to 4.70 *	Time	−0.93	0.48	−1.90 to 0.002
Pre Qx ROM	0.35	0.18	0.04 to 0.65 *	Baseline TSK11	0.77	0.35	0.15 to 1.39 *
Pre Qx Positive SETS	4.83	2.23	1.07 to 8.60 *	Age	0.15	0.16	−0.14 to 0.43
Age	0.37	0.23	−0.02 to 0.76	BMI	−0.63	0.40	−1.35 to 0.08
BMI	−0.35	0.51	−1.21 to 0.51				
**Random Effects**	*Std. Dev*			**Random Effects**	*Std. Dev*		
Subjects (intercept)	5.71			Subjects (intercept)	3.97		
**2**							
	HADS Anxiety (1)				HADS Anxiety (2)		
**Fixed Effects**	*Est.*	*SE*	*95% CI*	**Fixed Effects**	*Est.*	*SE*	*95% CI*
Intercept	3.59	3.01	−1.84 to 8.98	Intercept	−2.54	3.90	−9.48 to 4.40
Time	−0.84	0.19	−1.21 to −0.46 *	Time	−0.98	0.24	−1.45 to −0.51 *
Pre Qx HADS Anxiety	0.48	0.11	0.28 to 0.68 *	Baseline HADS Anxiety	0.64	0.13	0.41 to 0.87 *
Age	0.02	0.06	−0.08 to 0.12	Age	0.13	0.06	0.02 to 0.24 *
BMI	−0.01	0.13	−0.24 to 0.21	BMI	0.08	0.15	−0.18 to 0.34
**Random Effects**	*Std. Dev*			**Random Effects**	*Std. Dev*		
Subjects (intercept)	1.38			Subjects (intercept)	1.38		

1: (1) Forty-eight observations; (2) fifty-three observations; ROM: range of motion; Est.: estimate; SE: standard error; 95% CI: 95% confidence interval; Std Dev.: standard deviation; Pre Qx: pre-surgical; SETS: Stanford Expectations for Treatment Scale; BMI: body mass index; TSK11: Tampa Scale of Kinesiophobia. (*) *p*-values lower than 0.05. 2: (1) Fifty-eight observations; (2) fifty-four observations; HADS: Hospital Anxiety and Depression Scale; Est.: estimate; SE: standard error; 95% CI: 95% confidence interval; Std Dev.: standard deviation; Pre Qx: pre-surgical; BMI: body mass index; (*) indicates *p*-values lower than 0.05.

## Data Availability

The data presented in this study are available at the request of the corresponding author due to privacy and ethical reasons.
